# A Challenge for Diagnosing Acute Liver Injury with Concomitant/Sequential Exposure to Multiple Drugs: Can Causality Assessment Scales Be Utilized to Identify the Offending Drug?

**DOI:** 10.1155/2014/156389

**Published:** 2014-11-24

**Authors:** Roxanne Lim, Hassan Choudry, Kim Conner, Wikrom Karnsakul

**Affiliations:** Division of Pediatric Gastroenterology and Nutrition, Johns Hopkins University School of Medicine, Baltimore, MD 21287, USA

## Abstract

Drug-induced hepatotoxicity most commonly manifests as an acute hepatitis syndrome and remains the leading cause of drug-induced death/mortality and the primary reason for withdrawal of drugs from the pharmaceutical market. We report a case of acute liver injury in a 12-year-old Hispanic boy, who received a series of five antibiotics (amoxicillin, ceftriaxone, vancomycin, ampicillin/sulbactam, and clindamycin) for cervical lymphadenitis/retropharyngeal cellulitis. Histopathology of the liver biopsy specimen revealed acute cholestatic hepatitis. All known causes of acute liver injury were appropriately excluded and (only) drug-induced liver injury was left as a cause of his cholestasis. Liver-specific causality assessment scales such as Council for the International Organization of Medical Sciences/Roussel Uclaf Causality Assessment Method scoring system (CIOMS/RUCAM), Maria and Victorino scale, and Digestive Disease Week-Japan were applied to seek the most likely offending drug. Although clindamycin is the most likely cause by clinical diagnosis, none of causality assessment scales aid in the diagnosis.

## 1. Introduction

Acute drug-induced liver injuries (DILI) predominate (about 90% of cases) [1] and are classified into 3 categories [2], acute hepatocellular injury, acute cholestatic liver injury, and mixed pattern acute liver injury. When a single drug is involved, the diagnosis is relatively simple. The administration of multiple concomitant drugs however can pose a difficult implication for which a specific agent would be the cause of DILI. The administration of multiple concomitant drugs can, however, pose a conundrum as to which specific agent is the cause of DILI. Several algorithms/clinical scales have been developed to improve the accuracy, consistency, and objectiveness in identifying the offending drug for the causality assessment of adverse drug reactions. Examples include the Maria and Victorino scale [3] and Council for the International Organization of Medical Sciences/Roussel Uclaf Causality Assessment Method scoring system (CIOMS/RUCAM) scale [3–5], which are used primarily to quantify the strength of association between a liver injury and a particular drug being implicated. However, it must be emphasized that these diagnostic scales should not be substituted for clinical judgment.

We report a 12-year-old boy who received multiple antibiotics for the treatment of cervical lymphadenitis, retropharyngeal cellulitis, and developed signs and symptoms of cholestatic hepatitis. Causality assessment scales of adverse drug reactions including Council for the International Organization of Medical Sciences/Roussel Uclaf Causality Assessment Method scoring system (CIOMS/RUCAM), Maria and Victorino scale, and Digestive Disease Week-Japan (DDW-J) were utilized to identify the most probable offending drug.

## 2. Case Report

A previously healthy 12-year-old Hispanic boy presented with a history of sore throat and swelling in the right submandibular region without history of sick contact, travel, tick bites, or uncooked or raw food consumption. Amoxicillin was started to treat a probable streptococcal infection. Three days later he developed anorexia, dysphagia with liquids, and neck swelling with fever. He was brought to the Emergency Department of a nearby hospital and subsequently admitted. A computed tomography scan of the neck and chest showed a retropharyngeal fluid collection without features of an abscess or foreign body and lymphadenopathy throughout the neck and upper mediastinum. Ceftriaxone and vancomycin were started to treat diffuse facial and neck cellulitis for 3 days. While the neck swelling progressed, he was intubated. Ceftriaxone and vancomycin were discontinued, and ampicillin/sulbactam and steroid were started. The following day he was extubated due to improved neck swelling. Results from laboratory tests were all within normal limits except for mild anemia (hemoglobin 9.9 g/dL); however, liver function tests (LFT) were not performed at the time. Ampicillin/sulbactam and steroid were given for 2 days and discontinued. He was discharged home with a planned 10-day course of oral clindamycin 300 mg three times a day.

Over 5 days after the hospital discharge, he had developed fever, fatigue, headache, and dark urine. At the Emergency Department he had severe dehydration, fever (maximum temperature of 101°F), physical findings of mild hepatosplenomegaly, and right upper quadrant tenderness. LFT results included alanine aminotransferase (ALT), 406 IU/L (normal range 0–40); aspartate aminotransferase (AST), 98 IU/L (normal range 0–37); alkaline phosphatase (ALP), 404 IU/L (normal range 100–390); total bilirubin, 3.0 mg/dL (normal range 0.1–1.2); direct bilirubin, 2.7 mg/dL (normal range 0.0–0.4); prothrombin time (PT), 15.3 seconds; international normalized ratio (INR), 1.12; activated partial thromboplastin time (aPTT), 35.2 seconds. At this point, clindamycin was suspected as a cause of hepatic injury and ampicillin/sulbactam was started for 2 weeks because of the lower risk of hepatotoxicity. Infectious workups were all negative for hepatitis A, B, C, and E, Herpes, Epstein-Barr virus and cytomegalovirus viruses, Leptospira,* Bartonella henselae*, and blood culture. Three days after his admission in the Emergency Department, repeat LFTs were ALT, 232 IU/L; AST, 94 IU/L; ALP, 465 IU/L; total bilirubin (TB), 4.0 mg/dL; direct bilirubin (DB), 2.8 mg/dL. The patient was discharged home with clinical improvement.

Two days later at a follow-up with his pediatrician due to abdominal pain, enlarged liver was noted on examination and LFTs worsened: ALT, 152 IU/L; AST, 120 IU/L; ALP, 737 IU/L; TB, 8.8 mg/dL with peripheral eosinophilia with an absolute count of 900/*μ*L. Referral was made to pediatric liver specialist 5 days later when TB was at 9.9 mg/dL.

At the Pediatric Liver Center at Johns Hopkins Hospital the patient complained of chest pain, fatigue, pruritus, and a recent onset of acholic stools. Physical examination was unremarkable except icteric sclera and mild hepatomegaly. Further investigation was promptly started; ALT, 185 IU/L with upper limit normal of normal ULN at 34; AST, 208 IU/L; ALP, 812 IU/L; TB, 11.8 mg/dL; PT, 11 seconds; INR, 1.1; aPTT, 28.6 seconds; amylase, 26 U/L; lipase, 33 U/L; normal ammonia level, glucose, and thyroid function tests. Since serum ALT elevated > 3X ULN and serum bilirubin > 2X ULN and DILI is one of the possible liver injury causes, by Hy's Law he had the potential for development of acute liver failure. However his other liver synthetic function was normal and clinically he did not have hepatic encephalopathy. All-out efforts were made to look for every possible contributing cause of his acute liver injury. Specific liver investigations revealed normal ceruloplasmin, serum ferritin level, normal alpha-1 antitrypsin level, negative alpha-1 antitrypsin mutation analysis, and negative liver autoantibodies (antimitochondrial, antinuclear, antismooth-muscle, and antiliver-kidney microsomal antibodies).

Magnetic resonance cholangiopancreatography excluded abnormal gallbladder, intra- and extrahepatic bile duct system, and intrahepatic lesions as possible causes of cholestasis. Vanishing bile duct syndrome was suspected. At approximately day 48 after illness a percutaneous liver biopsy was performed. Histopathologic findings demonstrated moderate lobular cholestasis, mild patchy lobular chronic inflammation, and mild portal fibrosis without features of viral cytopathic effects, autoimmune hepatitis, bile duct injury or loss, and iron storage. Ursodeoxycholic acid was used to treat cholestasis (20 mg/kg/day). Fat soluble vitamins were supplemented. At six-month and 4-year follow-up after the onset of illness his liver chemistry profiles did not indicate ongoing cholestatic jaundice or hepatocellular injury.

**Figure 1:**
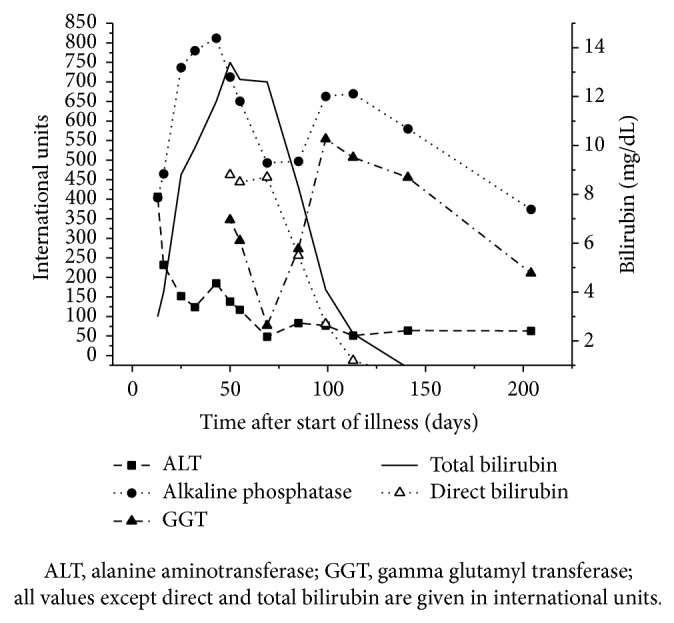
Evolution of laboratory values of liver function tests of patients with time.

Over the following 5 months, jaundice and pruritus gradually improved with a more than 50% improvement in transaminases and bilirubin values. Clindamycin-induced hepatic injury is highly suspected with evidence of previous reports in the literature, and using clinical judgement as displayed in Table 1, Figure 1 summarizes correlation between clinical/biochemical manifestations and drug administration, trends of transaminases and bilirubin values, and timeline of events. The question of drugs other than clindamycin possibly causing DILI in this case was raised for the appropriate information for future drug use in the future. Since multiple antibiotics were administered in the same temporal sequence, the probability of the hepatotoxicity being secondary to an adverse drug reaction from the other antibiotics administered prior to clindamycin was assessed using 3 scales (Table 2). Liver-specific causality assessment scales including CIOMS/RUCAM scoring system, Maria and Victorino scale, and DDW-J scale were applied to seek an offending drug but rated all the antibiotics as being equally “possible” and “probable” in causing the liver damage except amoxicillin showing lower score in Maria and Victorino clinical diagnostic scale.

## 3. Discussion

A period of 5–10 days after administration of multiple antibiotics, our patient had an acute presentation of cholestasis described by dark urine, icteric sclerae, and abnormal liver chemistry. Based on his history and physical examination, all known causes for cholestasis in the pediatric population were excluded by extensive investigations. Therefore, drug-induced liver injury (DILI) was proposed as a probable etiology. This was further supported as follows: (1) the development of cholestasis after the introduction of the antibiotics, (2) clinical and biochemical improvement after withdrawal of the drugs, (3) hepatotoxicity as a known adverse side effect of each of the antibiotics, and (4) histopathologic findings excluding other causes of cholestatic hepatitis.

DILI is a well-recognized problem that accounts for up to 10 percent of all adverse drug reactions. Two main mechanisms of DILI have been proposed: predictable injury (intrinsic hepatotoxins) and unpredictable injury (idiosyncratic reactions). In our case, an idiosyncratic reaction is likely to be the case. Many experts would suggest that the liver injury could have been primarily caused by clindamycin and that subsequent medications played no role in the presentation. Although fever, abdominal pain, and hepatomegaly followed 5–10 days after administration of several antibiotics, clindamycin is the immediate agent that started right before the presentation. The causality assessment scales for DILI were used as tools, for this reason, to give more clues as to which antibiotic would more likely be the offending agent.

In order to facilitate causality assessments for DILI, several methods have been developed, including expert judgement, probabilistic approaches, and algorithms/scales [6–8]. The latter can be divided into general and liver-specific scales. As strength in general any of standardized causality assessment scales enhance objectivity in case assessments, grade the strength of probability in broad categories, and can provide warning signs for drug regulatory measurements. However these scales are often complex and time consuming to operate. They do not provide a certain diagnosis of DILI. Each of these has its own strengths and weaknesses. For example, although the CIOMS/RUCAM scale is cumbersome and lacks intra- and interrater reliability, it is however the preferred method as a result of simple and practical use [9–11]. Absolute agreement between the scales could be low [3]. All scales are not designed to evaluate DILI when concomitant drugs are used to solve this particular problem. Therefore the scales do not replace clinical judgement. Exposure to multiple drugs during the same period is a challenging factor in identifying a single agent as a probable offending drug and poses a dilemma for future recommendation for drug use.

At the time of consultation on this case, we recommended holding off the use of clindamycin unless there was no available option for the prospective infection patient might have. Unfortunately, even for IgE mediated drug allergy, testing is very limited, and for this kind of situation there is not any commercial testing that would be helpful given most likely idiosyncratic mechanism in nature. When we look at cases like this, all that can be done is to consider which drugs are more likely to have this type of side effect and then proceed cautiously. However for an idiosyncratic reaction, it is unpredictable as the same drug(s) may not do the same thing in the future. DDW-J was the recently proposed scale in Japan which was modified from CIOMS adding in vitro drug lymphocyte stimulation test (DLST) [12]. The test demonstrates an immunological mechanism for the DILI by demonstrating the existence of a subset of T lymphocytes which recognize and are activated by the drug [13]. Recent findings on HLA allele associations with DILI via adaptive immune response suggested the benefit of DLST utilization [13]. DLST was not performed in their patient as it is not currently commercially available in the United States. The pros of DLST are that we will have a clue which drugs would be the prime candidate causing the reaction and the information could be used as a guide for an avoidance of the suspected drug. In theory DLST is the ideal and objective way to understand the immunologic response to the offending drug; however, many reactions are idiosyncratic, so the same drug(s) may not cause the same reaction in the future. As demonstrated in Table 2, each of the antibiotics administered has an almost equal probability of causing DILI. When we assessed the causality by different scales for ceftriaxone, vancomycin, clindamycin, and ampicillin/sulbactam (Table 2), scores for each were in similar category as a “possible” or “probable” cause of liver injury.

A search of the literature revealed that all five antibiotics have been known to be implicated in DILI. Most of isolated adult cases, however, may not reflect on the clinical aspect in children [14]. Although the reports in pediatric cases were limited, there was a recent pediatric report comparing several antibiotic uses with a focus on hepatic injury [14].

Maraqa et al. reported a 13-year-old child with clindamycin related DILI. The time to onset was 17 days and time to resolution was 10 days [15]. As for LiverTox database it only speaks of case reports in adults for hepatic toxicity from clindamycin. The rest of journal articles only relate hepatotoxicity to clindamycin in adult patients. A 42-year-old woman developed fatigue, nausea, vomiting, anorexia, pruritus, and jaundice 6 days after administration of the last clindamycin dose for a dental infection [16]. Transaminases were markedly elevated and liver biopsy revealed centrilobular and portal cholestatic hepatitis without fibrosis or necrosis. There is also another case of a 67-year-old man who received a 10-day course of oral clindamycin for a skin abscess. One week after the last clindamycin dose, he developed icterus accompanied by pale stools, dark urine, and pruritus [17]. Liver biopsy revealed marked cholestasis, portal inflammation, bile duct injury, and ductopenia. A second biopsy five months after the first one showed resolved cholestasis but persistent ductopenia.

Molleston et al. reported two cases of DILI secondary to amoxicillin use among pediatric patients in the DILIN prospective study [18]. Kim et al. presented a case of a 39-year-old woman who developed cholestatic hepatitis with bile duct damage and hepatocellular injury eight weeks after initiation of amoxicillin treatment for abdominal actinomycosis [19] and became asymptomatic fourteen weeks after drug discontinuation.

Several articles have been published in the literature documenting the association between ceftriaxone and DILI. Peker et al. reported a 12-year-old boy who complained of weakness 3 days and had elevated transaminases 6 days after ceftriaxone was given for tonsillitis [20]. Transaminases eventually returned to baseline within 10 weeks after discontinuing the drug. Bickford and Spencer described a case of a 53-year-old man who had elevated total and direct bilirubin levels after a week therapy of ceftriaxone [21]. Discontinuing ceftriaxone led to normalization of the LFTs within 2 weeks.

Chen et al. conducted a meta-analysis of 20 published randomized controlled trials involving 7419 patients [22]. An increased incidence of hepatic events, specifically elevated serum aminotransferase levels, was observed in patients receiving vancomycin (6.8%) compared to those who were not (3.9%). The majority of events were mild to moderate in nature and progressive or severe DILI has not been associated with vancomycin use.

Ampicillin/sulbactam, in a rare incident, was reported in a 74-year-old man with Hodgkin's disease in remission, who developed a 3-month period of cholestasis after a week treatment with ampicillin/sulbactam 750 mg twice daily for sore throat [23]. Abnormal liver enzymes prompted liver biopsy showing diffuse canalicular and mild hepatocellular cholestasis, mild and mixed inflammation in the portal area, and diffuse necroinflammatory areas in the liver parenchyma. Liver function tests eventually normalized 7 months after discontinuing the drug.

In DILI cases with concomitant or sequential drug exposure the CIOMS/RUCAM scale may not be able to differentiate between offending drugs and would require individual assessments for each of the drugs. These causality assessment scales disregard differences in metabolic pathways utilized by concomitant drugs and potential pharmacokinetic drug-drug or drug-disease interactions [3]. DLST in DDW-J scale on any drugs could predict the hepatotoxic potential of such a drug making it the prime candidate causing DILI. CIOMS has many shortcomings which render it inaccurate in assessing causality in a multipharmacy situation. A consensus on criteria for excluding nondrug-related cases will establish a standardized evidence based database of drugs causing DILI. This knowledge would allow physicians to apply a uniform scoring system in the sections of “concomitance therapy” and previous information on hepatotoxicity [24]. A “multihit” process could explain idiosyncratic drug reaction in this patient as a result from a succession of events or exposure to multiple drugs [25]. It is unlikely that genetic variants in isoenzymes or cytochrome 450 pathway alone would predispose to severe hepatotoxicity from toxic byproducts given that severe liver toxicity is a rare event. In addition there could be suppressor or attenuator pathways which could play a role in idiosyncratic hepatotoxicity.

In summary, we report on a case of probable DILI with concomitant use of several antibiotics. None of the known causality assessment scoring systems was developed for identifying an offending drug for DILI cases with concomitant drug use in the same temporal sequence. Close monitoring of liver functions could eliminate certain drugs as offending agents if an abnormal LFT is present before and after certain drugs. Obviously this still continues to pose us a challenge in determining which of the drugs is/are allowed to be utilized in this child in the future. Whenever suspected, the offending drug should be discontinued immediately as complete recovery is still possible with prompt drug discontinuation. A different model using knowledge of drug metabolism and interaction via genetic variant isoenzymes, cytochrome P450 pathway, and application of in vitro DLST could be considered to aid in identifying offended drug causing DILI.

## Figures and Tables

**Table 1:** Correlation between clinical and biochemical manifestations and drug administration.

Time from onset of illness	Signs/symptoms	Drug exposure	Laboratory values				
			ALT	ALP	Total bilirubin	Direct bilirubin	Misc.
−5	Sore throat	Amoxicillin started					
0	Fever, anorexia, dysphagia, neck swelling, and cervical lymphadenopathy	Ceftriaxone and vancomycin started					
3–5	Worsening of neck swelling	Ceftriaxone and vancomycin discontinued, and ampicillin/sulbactam and steroid started					
5–10	Diarrhea	Ampicillin/sulbactam and steroid discontinued, and clindamycin and probiotics started					
10	Fever, headache, dizziness, chest pain on coughing, and dark urine. Hepatosplenomegaly, RUQ tenderness	Clindamycin switched to ampicillin/sulbactam	406	404	3.0	2.7	
16–23	Generalized maculopapular rash and pruritus	Ampicillin/sulbactam	152	737	8.8		
25–30	Abdominal pain, vomiting. Hepatomegaly	Ampicillin/sulbactam	124	780	9.9		AST 130
48	Chest pain, fatigue, pruritus, acholic stools, and jaundice		138	713	13.4	8.8	Cholesterol 1044, GGT 347,
53	Increasing pruritus, anorexia		117	651	12.7	8.5	GGT 294
67	Anorexia, pruritus		48	493	12.6	8.7	GGT 77
83	Anorexia		83	497	8.3	5.5	GGT 273
111	No anorexia		51	670	2.3	1.2	GGT 507

Time is in days. ALP = alkaline phosphatase, units used for ALT, ALP, and GGT used are IU. Bilirubin and cholesterol are displayed in mg/dL. Misc. = miscellaneous lab values.

**Table 2:** Comparison of three liver-specific causality assessment scales on multiple sequential drug exposure.

Causality assessment scales and criteria	CIOMS/RUCAM	Maria and Victorino clinical diagnostic scale	(DDW-J) scale
Chronological criteria			
From drug intake until onset	Score range: +1 to +2 (i) 5–90 days: +2 (ii) <5 or >90 days: +1	Score range: +1 to +3 (i) 4 d–8 wks: +3 (ii) <4 d or >8 wks: +1	Score range: +1 to +2 (i) 5–90 d/1–90 days: +2 (ii) <5 or >90 d/>15 days: +1
Withdrawal until onset	Score range: 0 to +1 (i) ≤30 d: +1 (ii) 0–29 d: 0	Score range: −3 to +3 (i) 0–7 d: +3 (ii) 8–15 d: 0 (iii) >15 d: –3	Score range: 0 to +1 (i) ≤30 d: +1 (ii) >30 d: 0

Course of the reaction	Score range: −2 to +3 Improvement in 180 days: (i) >50%: +2 (ii) <50%: +1 (iii) Lack of info or no improvement: +0	Score range: 0 to +3 (i) <6 mths (cholestatic/mixed) or <2 mths (hepatocellular): +3 (ii) >6 or 2 mths: +0	Score range: −2 to +3 After cessation of drug Difference in ALP peak and ULN (i) not applicable: +3 (ii) decrease in liver enzymes ≥50% in 180 d: +2 decrease <50% in 180 d: +1 (iii) no information/ persistence/increase: +0 (iv) N/A: −2

Extrahepatic manifestations	N/A	Score range: 0 to +3 (rash, fever, arthralgia, eosinophilia >6%, and cytopenia) (i) ≥4: +3 (ii) 2 or 3: +2 (iii) 1: +1 (iv) None: 0	Score range: 0 to+1 Eosinophilia (≥6%) (i) present: +1 (ii) absent: +0

Risk factors	Score range: 0 to +2 (i) Age ≥55: +1 (ii) Alcohol or pregnancy: +1	N/A	Score range: 0 to +1 (i) alcohol/pregnancy: +1

Concomitant therapy	Score range: −3 to 0 Time to onset: (i) incompatible: +0 (ii) compatible but with unknown reaction: −1 (iii) compatible but known reaction: −2 (iv) role proved in this case: −3 (v) none or information not available: +0	N/A	N/A

Exclusion of other causes	Score range: −3 to +2 (i) ruled out: +2 (ii) “possible” to “not investigated”: −2 to +1 (iii) probable: −3	Score range: −3 to +3 (i) complete: +3 (ii) partial: +0 (iii) possible alt cause: −1 (iv) probable alt cause: −3	Score range: −3 to +2 (i) ruled out: +2 (ii) 6 causes of Group I ruled out: +1 (iii) 5/4 causes of Group I ruled out: +0 (iv) <4 causes of Group I ruled out: −2 (v) nondrug cause highly probable: −3

Previous information or known reaction	Score range: 0 to +2 Reaction: (i) unknown: +0 (ii) published but unlabelled: +1 (iii) labeled in the product's characteristics: +2	Score range: −3 to +2 (i) yes: +2 (ii) no (drug marketed for ≤5 yrs): +0 (iii) no (drug marketed for <5 yrs): −3	Score range: 0 to +1 (i) reaction labelled in product characteristics or published: +1 (ii) reaction unknown: +0

Rechallenge	Score range: −2 to +3 (i) positive: +3 (ii) compatible: +1 (iii) negative: −2 (iv) Not available/interpretable: +0 (v) plasma conc. of drug toxic: +3	Score range: 0 to +3 (i) positive: +3 (ii) negative/absent: +0	Score range: 0 to +3 (i) ALP/TB ≥ 2x with drug alone: +3 (ii) ALP/TB ≥ 2x with drug already given at time of 1st reaction: +1 (iii) ALP/TB increases but <*N* − 2: −2 (iv) Other situations: +0

DLST	N/A	N/A	Score range: 0 to +2 DLST (i) positive: +2 (ii) semipositive: +1 (iii) negative/unavailable: +0

Scores interpretation	(i) >8 points: definite (ii) 6–8 points: probable (iii) 3–5 points: possible (iv) 1-2 points: unlikely (v) <0 points: excluded	(i) >17 points: definite (ii) 14–17 points: probable (iii) 10–13 points: possible (iv) 6–9 points: unlikely (v) <6 points: excluded	(i) >4 points: definite (ii) 3-4 points: probable (iii) <3 points: unlikely

Our case scores			
Amoxicillin	7 probable	10 possible	7 high possibility
Ceftriaxone	7 probable	13 possible	7 high possible
Vancomycin	7 probable	13 possible	7 high possibility
Ampicillin/sulbactam	7 probable	13 possible	7 high possibility
Clindamycin	7 probable	13 possible	7 high possibility

CIOMS, Council for the International Organization of Medical Sciences; DDW-J, Digestive Disease Week-Japan; DLST, Drug lymphocyte stimulation test; N/A, not available; d, days.

## References

[1] Zimmerman H. J. (1999). *Hepatotoxicity: The Adverse Effects of Drugs and Other Chemicals on the Liver*.

[2] Benichou C., Benhamou J. P., Danan G. (1990). Criteria of drug-induced liver disorders. *Journal of Hepatology*.

[3] Garcia-Cortes M., Stephens C., Lucena M. I., Fernandez-Castañer A., Andrade R. J. (2011). Causality assessment methods in drug induced liver injury: strengths and weaknesses. *Journal of Hepatology*.

[4] Danan G., Benichou C. (1993). Causality assessment of adverse reactions to drugs—I: a novel method based on the conclusions of international consensus meetings: application to drug-induced liver injuries. *Journal of Clinical Epidemiology*.

[5] Benichou C., Danan G., Flahault A. (1993). Causality assessment of adverse reactions to drugs—II: an original model for validation of drug causality assessment methods: case reports with positive rechallenge. *Journal of Clinical Epidemiology*.

[6] Larrey D. (2000). Drug-induced liver diseases. *Journal of Hepatology*.

[7] Gunawan B. K., Kaplowitz N. (2007). Mechanisms of drug-induced liver disease. *Clinics in Liver Disease*.

[8] Zapater P., Such J., Pérez-Mateo M., Horga J. F. (2002). A new poisson and Bayesian-based method to assign risk and causality in patients with suspected hepatic adverse drug reactions: a report of two new cases of ticlopidine-induced hepatotoxicity. *Drug Safety*.

[9] Hayashi P. H. (2009). Causality assessment in drug-induced liver injury. *Seminars in Liver Disease*.

[10] Shapiro M. A., Lewis J. H. (2007). Causality assessment of drug-induced hepatotoxicity: promises and pitfalls. *Clinics in Liver Disease*.

[11] García-Cortés M., Lucena M. I., Pachkoria K., Borraz Y., Hidalgo R., Andrade R. J. (2008). Evaluation of naranjo adverse drug reactions probability scale in causality assessment of drug-induced liver injury. *Alimentary Pharmacology and Therapeutics*.

[12] Takikawa H., Takamori Y., Kumagi T., Onji M., Watanabe M., Shibuya A., Hisamochi A., Kumashiro R., Ito T., Mitsumoto Y., Nakamura A., Sakaguchi T. (2003). Assessment of 287 Japanese cases of drug induced liver injury by the diagnostic scale of the International Consensus Meeting. *Hepatology Research*.

[13] Watkins P. B. (2009). Biomarkers for the diagnosis and management of drug-induced liver injury. *Seminars in Liver Disease*.

[14] Serranti D., Montagnani C., Indolfi G., Chiappini E., Galli L., de Martino M. (2013). Antibiotic induced liver injury: what about children?. *Journal of Chemotherapy*.

[15] Maraqa N. F., Gomez M. M., Rathore M. H., Alvarez A. M. (2002). Higher occurrence of hepatotoxicity and rash in patients treated with oxacillin, compared with those treated with nafcillin and other commonly used antimicrobials. *Clinical Infectious Diseases*.

[16] Aygün C., Kocaman O., Gürbüz Y., Şentürk Ö., Hülagü S. (2007). Clindamycin-induced acute cholestatic hepatitis. *World Journal of Gastroenterology*.

[17] Altraif I., Lilly L., Wanless I. R., Heathcote J. (1994). Cholestatic liver disease with ductopenia (vanishing bile duct syndrome) after administration of clindamycin and trimethoprim-sulfamethoxazole. *The American Journal of Gastroenterology*.

[18] Molleston J. P., Fontana R. J., Lopez M. J., Kleiner D. E., Gu J., Chalasani N. (2011). Characteristics of idiosyncratic drug-induced liver injury in children: results from the DILIN prospective study. *Journal of Pediatric Gastroenterology and Nutrition*.

[19] Kim J. S., Jang Y. R., Lee J. W., Kim J. Y., Jung Y. K., Chung D. H., Kwon O. S., Kim Y. S., Choi D. J., Kim J. H. (2011). A case of amoxicillin-induced hepatocellular liver injury with bile-duct damage. *The Korean Journal of Hepatology*.

[20] Peker E., Cagan E., Dogan M. (2009). Ceftriaxone-induced toxic hepatitis. *World Journal of Gastroenterology*.

[21] Bickford C. L., Spencer A. P. (2005). Biliary sludge and hyperbilirubinemia associated with ceftriaxone in an adult: case report and review of the literature. *Pharmacotherapy*.

[22] Chen Y., Yang X. Y., Zeckel M., Killian C., Hornbuckle K., Regev A., Voss S. (2011). Risk of hepatic events in patients treated with vancomycin in clinical studies: a systematic review and meta-analysis. *Drug Safety*.

[23] Köklü S., Köksal A. Ş., Asil M., Kiyici H., Çoban Ş., Arhan M. (2004). Probable sulbactam/ampicillin-associated prolonged cholestasis. *Annals of Pharmacotherapy*.

[24] Fontana R. J., Seeff L. B., Andrade R. J., Björnsson E., Day C. P., Serrano J., Hoofnagle J. H. (2010). Standardization of nomenclature and causality assessment in drug-induced liver injury: summary of a clinical research workshop. *Hepatology*.

[25] Lee W. M. (2003). Drug-induced hepatotoxicity. *The New England Journal of Medicine*.

